# Indirect-Oscillation Sign Suggesting Infective Endocarditis on the Routine Chest CT

**DOI:** 10.3390/jcdd11100335

**Published:** 2024-10-21

**Authors:** Min Ji Son, Seung Min Yoo, Hwa Yeon Lee, Charles S. White

**Affiliations:** 1 Department of Radiology, CHA University Bundang Medical Center, Bundang 13496, Republic of Korea; smj3006@naver.com; 2 Radiology, Smile Radiologic Clinic, Seoul 03636, Republic of Korea; hynlee1@hanmail.net; 3 Department of Radiology, University of Maryland, Baltimore, MD 21201, USA; cswhite999@gmail.com

**Keywords:** infective endocarditis, chest CT, splenic or renal infarction

## Abstract

Routine chest CT is not essential for the diagnostic workup for infective endocarditis (IE), but this type of study may be the initial imaging modality in the evaluation of patients ultimately proven to have IE who present to the emergency department with nonspecific clinical symptoms. Although routine chest CT cannot directly assess valvular oscillating motion due to the lack of cine images, we hypothesized that a combination of elongated nodular valve thickening and abnormal orientation to the normal valve with a blind end on routine CT (indirect-oscillation sign) might suggest movable vegetation indirectly. To evaluate this possibility, we studied 27 patients with IE and 35 controls who underwent both routine chest CT and echocardiography. CT scanning was initiated following a delay of 60–80 s after the administration of the contrast medium. Two cardiothoracic radiologists retrospectively analyzed the CT images to assess the indirect-oscillation sign with consensus. The sensitivity, specificity, positive predictive value, and negative predictive value of the indirect-oscillation sign on routine chest CT were 29.6% (8/27), 100% (35/35), 100% (8/8), and 64.8% (35/54), respectively. Although uncommon, the presence of the indirect-oscillation sign involving the aortic or mitral valve on routine chest CT is a suggestive finding for IE.

## 1. Introduction

Infective endocarditis (IE) is defined as an infection of the endocardium, heart valves, or intra-cardiac devices, which can be fatal if not adequately treated [1,2,3]. IE is mainly caused by bacteria entering the blood stream from various sites of primary infection (e.g., skin, urogenital infection, infected indwelling catheter or cardiac devices, dental infection). The most frequently implicated organism is staphylococcus, followed by streptococcus, enterococcus, and others. Infective endocarditis often occurs in patients with predisposing cardiac risk factors, such as prosthetic cardiac valves, congenital heart disease, and valvular heart disease. A flow jet or turbulent blood flow produced by underlying cardiac pathology may result in endocardial injury or thrombus formation, leading to bacterial adherence and the subsequent development of IE [4]. The diagnosis of IE is based on the modified Duke criteria [5]. The modified Duke criteria consist of two major criteria (specifically, findings that are highly suggestive of IE based on blood culture and a positive imaging test) and five minor criteria. These are the presence of a predisposing heart condition such as valvular or congenital heart disease, fever (>38 °C), arterial embolism such as splenic infraction, immunologic phenomena such as glomerulonephritis or Osler’s nodes, and an equivocal blood culture result that does not fit the major criteria. Definite IE is defined by the presence of two major criteria, one major and three minor criteria, or five minor criteria. Possible IE is considered when one major and one minor criteria or three minor criteria are present [6]. Transthoracic echocardiography (TTE) is the choice of imaging modality for the diagnosis of IE, but nondiagnostic results may occur on TTE. Transesophageal echocardiography (TEE) has better spatial resolution and is particularly useful in patients with a clinical suspicion of IE [7,8]. However, echocardiography has several shortcomings such as its dependence on user experience, a narrow window, and diminished accuracy in patients with severe valve calcification or a prosthetic valve. Recently, ECG-gated cardiac computerized tomography (CT) has emerged as a complementary tool for echocardiography in patients with suboptimal results on TTE caused by severe valve calcification or those who have contraindications for TEE [9,10,11,12,13,14]. In contrast, there have been no prior studies that have evaluated the diagnostic accuracy of non-ECG gated routine enhanced chest CT for the diagnosis of IE, mainly due to concerns about inherent motion artifact of the valve due to the lack of ECG gating and its relatively lower temporal resolution compared to ECG-gated cardiac CT. More recently, it has been recognized that the development of wide-detector CT and the improved spatial and temporal resolutions of non-ECG-gated enhanced chest CT (routine chest CT) may mitigate motion artifacts [15,16]. Furthermore, routine chest CT has the potential to identify extracardiac findings caused by IE including splenic or renal infarction because the field of view on routine chest CT typically includes the upper portion of the spleen and parts of both kidneys. The authors recently encountered multiple patients who ultimately proved to have IE and in whom non-ECG-gated enhanced chest CT (i.e., routine chest CT) had been performed as the first-line imaging modality in the emergency department (ED). The CT scans were obtained in the context of a nonspecific clinical presentation (i.e., nonspecific fever or acute dyspnea), but the diagnosis of IE was not suggested on the initial CT interpretation. In a substantial proportion of these cases, suggestive findings for IE were identified on a retrospective assessment of these CTs. Thus, the purpose of this study is to evaluate the accuracy of routine enhanced chest CT for the diagnosis of IE.

## 2. Materials and Methods

### 2.1. Patients

**Figure 1:**
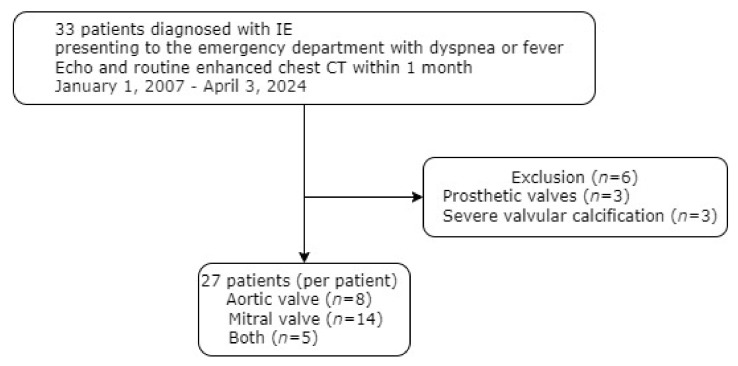
Flow chart of this study.

This study was approved by the Institutional Review Board (28 December 2023), and informed consent was waived due to its retrospective design, which was categorized as not more than minimal risk to participants. This study was performed in a university hospital with 1000 beds. This study included 33 patients who presented to the ED with fever or dyspnea, and who received a final diagnosis of IE involving the aortic or mitral valve between 1 January 2007 and 3 April 2024 (Figure 1). In each patient, routine enhanced chest CT and echocardiography were performed within one month of each other. Six patients were excluded from this study due to the presence of prosthetic valves (*n* = 3) or severe valvular calcification (*n* = 3). Specifically, the patients were excluded because severe metallic or blooming artifacts may interfere with the evaluation of potential vegetations on a non-ECG-gated routine chest CT. Following these exclusions, 27 patients with IE comprised the final study group. Twelve patients were confirmed to have IE by surgery. In the remaining 15 patients, IE was diagnosed based on the results of echocardiography and clinical findings [8]. During the same period, a randomly selected group of 35 patients who presented to the ED with nonspecific fever or dyspnea were included as a control group.

### 2.2. CT and Echocardiographic Technique

Non-ECG-gated enhanced chest CT (i.e., routine chest CT) was performed using 64-slice multi-detector CT (MDCT) scanners based in the ED (LightSpeed VCT, GE Healthcare, Milwaukee, WI, USA). The parameters for the chest CT scans were as follows: 120 kV, 200 mA, gantry rotation time of 0.45 s, temporal resolution of 0.23 s, collimation of 0.625 mm, increment of 1.5 mm, and reconstructions at 3 mm. Intravenous injection of 60 to 120 mL of Ioversol (Optiray 320 mg/mL, Tyco Healthcare, Montreal, QC, Canada) was administered via the antecubital vein at a flow rate of 3–5 mL/s based on the patient’s body mass index. CT scanning was started following a delay of 60–80 s after the administration of the contrast medium. Scanning coverage extended from the thoracic inlet to the adrenal glands. Standard 2D and Doppler measurements were interpreted using a standard commercially available ultrasound machine (Vivid E95 color Doppler ultrasound M5Sc-D probe; GE Medical Systems, Chicago, IL, USA; Philips Epiq 7 color Doppler ultrasound X5-1 probe; Philips Healthcare, Best, The Netherlands) with a frequency of 2.5–3.5 MHz, by one of six board-certified cardiologists.

### 2.3. Image Analysis

Two cardiothoracic radiologists retrospectively analyzed the CT findings on the routine chest CT (including axial, coronal, and sagittal images) in the absence of clinical information, focusing on the following: image quality, presence or absence of nodular valve thickening, presence or absence of complications of IE such as perivalvular abscess or pseudoaneurysm, and extracardiac findings of IE such as splenic or renal infarction.

CT image quality was categorized into two groups: diagnostic image quality with minimal or mild blurring versus nondiagnostic image quality in which artifacts could not be reliably excluded due to moderate to severe blurring. In the per-valve analysis, each valve showing normal or nondiagnostic image quality was counted as negative for the identification of IE to reflect real-world CT reading practice. With respect to an abnormal result, nodular valve thickening greater than 3 mm was considered as positive for IE. In the per-patient analysis for the identification of IE on the routine chest CT, the presence of nodular thickening involving at least one valve (either aortic or mitral valve or both valves) was considered positive in patients with IE.

**Figure 2:**
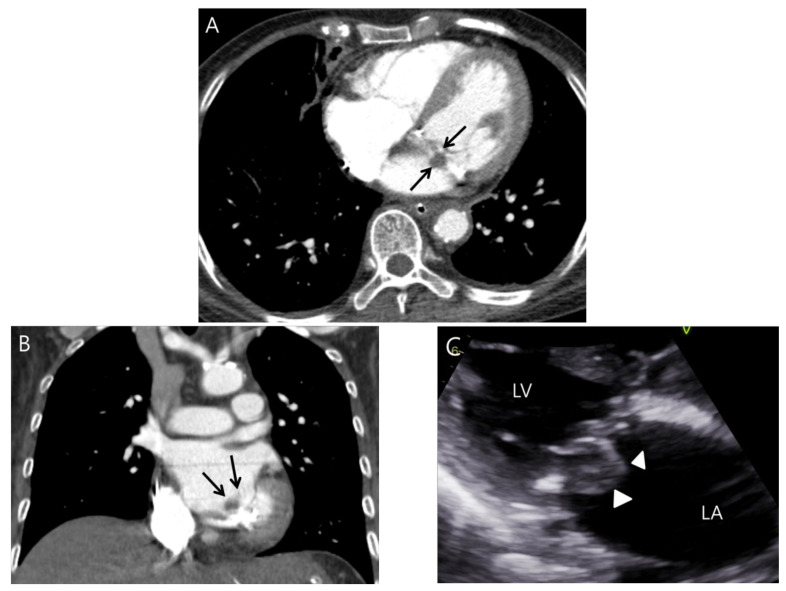
A case of nonelongated nodular mitral valve thickening observed on routine chest CT. A 69-year-old woman presented with fever. Axial (**A**) and coronal images (**B**) from routine chest CT show nonelongated nodular thickening (arrows) at the mitral valve. (**C**) Transthoracic echocardiography (TTE) demonstrates an echogenic vegetation with elongated appearance (arrowheads) on the mitral valve. LA and LV indicate left atrium and ventricle, respectively.

**Figure 3:**
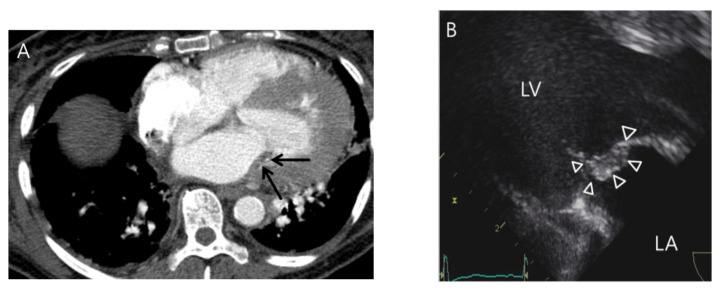
A case of elongated nodular thickening of the mitral valve without an indirect-oscillation sign demonstrated on routine chest CT. A 62-year-old woman presented with fever and dyspnea. (**A**) Elongated nodular thickening (arrows) at the lateral portion of the mitral valve was noted on an axial CT image. However, an indirect-oscillation sign was considered absent in this patient due to its adherent appearance to the mitral valve and the lack of a blind end. (**B**) The CT finding was confirmed to be a vegetation (arrowheads) with elongated appearance on subsequent transesophageal echocardiography.

Nodular valve thickening on routine chest CT was further classified as nonelongated (Figure 2) versus elongated nodular (Figure 3) based on empirical experience that the latter shape on CT might favor a vegetation over the former. Valve thickening was defined as elongated if the maximal length of the nodular thickening was greater than three times its thickness. For quantification, nodular thickening was measured using the mean diameter of the maximal length and the right-angle thickness on CT, and was compared to the size found on the echocardiography. 

**Figure 4:**
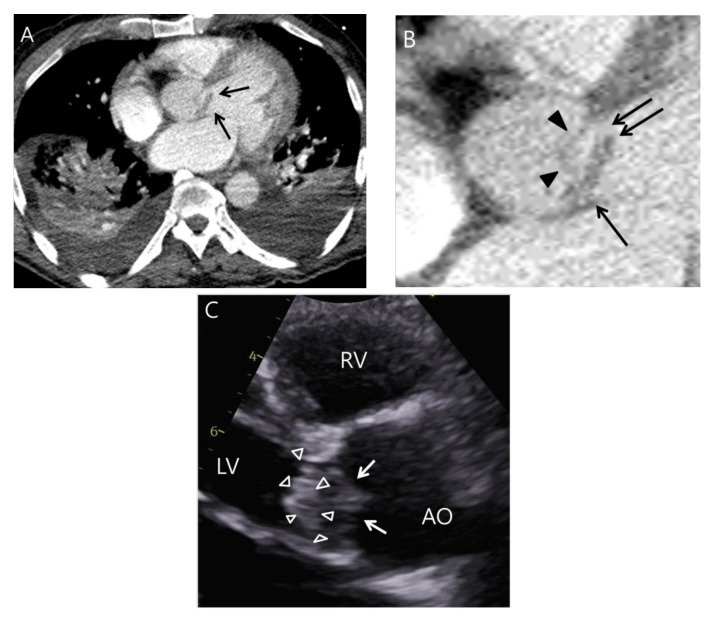
A case of the indirect-oscillation sign on routine chest CT. A 62-year-old man presented to the ED with acute dyspnea. (**A**) Note the area of elongated nodular thickening attached to the aortic valve on an axial CT image. (**B**) On the magnified view, the elongated nodular thickening is clearly different than the normal aortic valve cusp (arrowheads) because it has a blind end (double arrows) with an abnormal orientation. This CT finding is considered indicative of IE on routine chest CT (i.e., indirectly reflecting oscillation). (**C**) Subsequent transthoracic echocardiography (TTE) revealed an echogenic vegetation with elongated appearance (arrowheads) attached to the aortic valve (arrows). LV, RV, and AO indicate left ventricle, right ventricle, and aorta, respectively.

**Figure 5:**
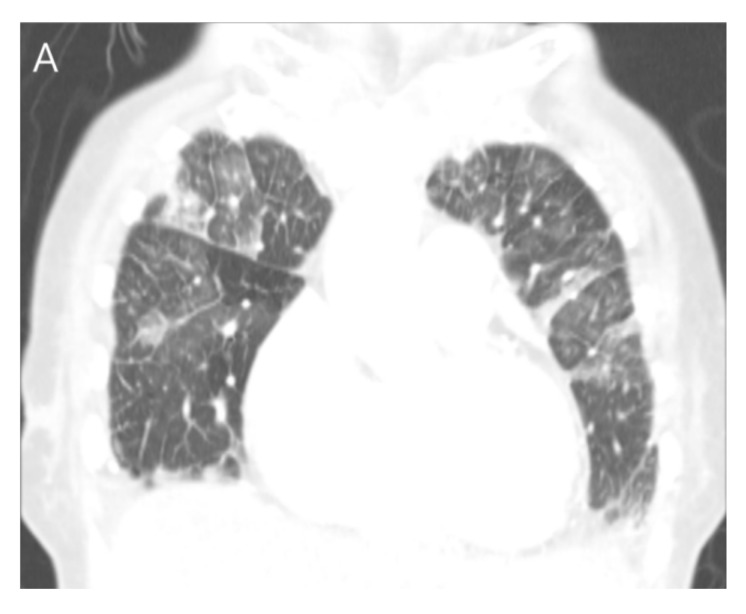

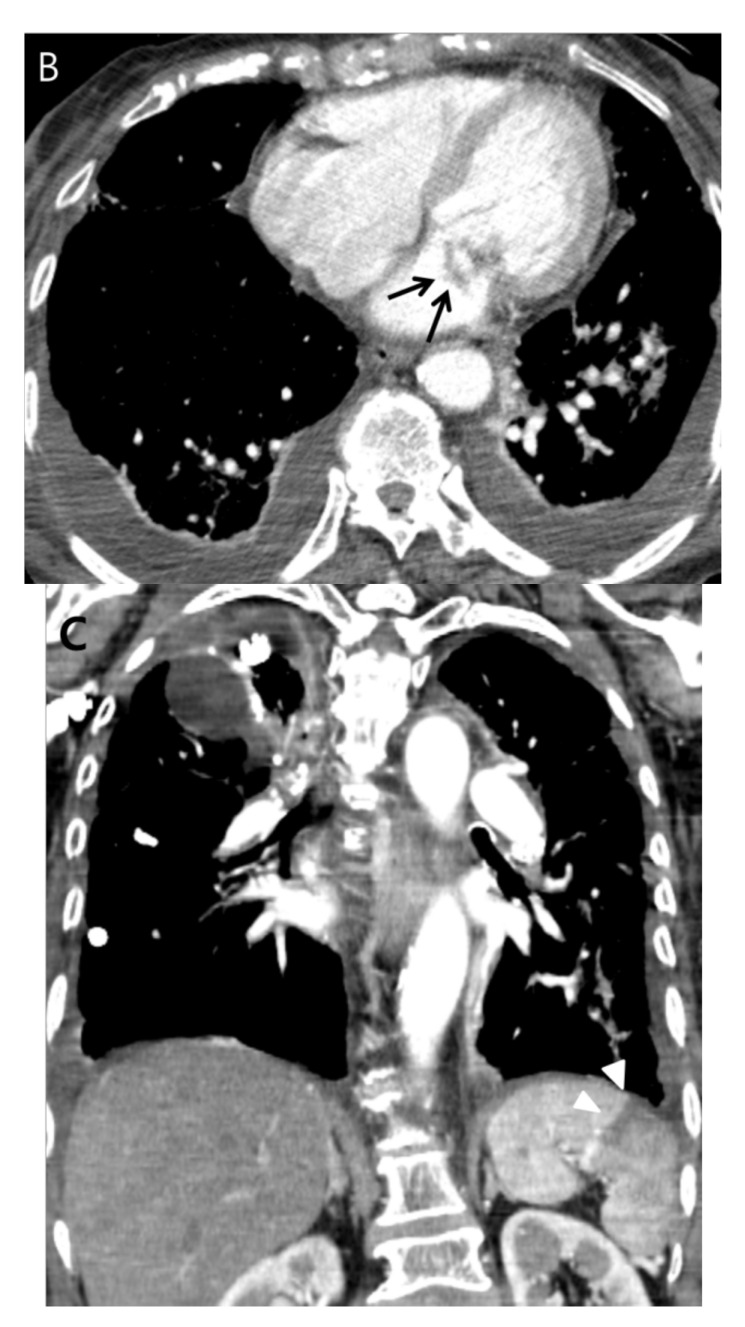
A case of an indirect-oscillation sign with splenic infarction on CT. IE involves the mitral valve on routine chest CT in a 75-year-old female patient. (**A**) Note the interlobular septal thickening and ground glass opacities in both lungs, suggesting the presence of pulmonary edema on a coronal CT image. (**B**) On an axial CT image, elongated nodular thickening with a blind end (double arrows), an abnormal orientation from the normal mitral valve, and attachment to the mitral valve (i.e., indirect-oscillation sign) may indicate the presence of vegetation (arrows). (**C**) Note the splenic infarction on a coronal CT image (arrowheads).

As routine chest CT cannot directly detect the presence of an oscillating vegetation due to the lack cine images, the authors hypothesized that a combination of the following routine chest CT features (deemed the “indirect-oscillation sign”) might indirectly suggest an oscillating vegetation: (1) elongated valve thickening; (2) abnormal orientation from the normal mitral or aortic valve with a blind end (Figure 4 and Figure 5). It seems reasonable that such a configuration on CT may be a marker for oscillating motion when the heart contracts. The authors also assessed for perivalvular abscess, or pseudoaneurysm, as defined by a prior study [13]. Splenic or renal infarction on routine chest CT was defined as follows: a wedge-shaped low attenuation in the spleen or kidney with a wider base on CT (Figure 5). Discrepancies between readers were resolved through consensus.

### 2.4. Statistical Analysis

Statistical analysis was performed using SPSS 27 (SPSS; Chicago, IL, USA). Fisher’s exact test was used for variables such as elongated valve thickening, the indirect-oscillation sign, and the combination of indirect-oscillation sign with splenic or renal infarction. A chi-square test was employed for variables such as nonelongated valve thickening and splenic or renal infarction, which are categorical variables. Student’s *t*-test was used for continuous variables with a normal distribution and equal variance. A statistically significant difference was defined as *p* < 0.05.

The sensitivity, specificity, negative predictive value (NPV), and positive predictive value (PPV) in diagnosing IE were calculated based on the following formulas: sensitivity (TP/(TP + FN)), specificity (TN/(TN + FP)), positive predictive value (TP/(TP + FP)), and negative predictive value (TN/(TN + FN)). TP, TN, FP, and FN indicate true positive, true negative, false positive, and false negative, respectively [17].

Interobserver agreement concerning the positive CT findings of IE on CT scans was evaluated using Cohen’s kappa coefficient.

## 3. Results

With respect to patient characteristics, there was a significant difference in the incidence of bicuspid aortic valve in patients with IE as compared to the control group. Otherwise, there was no significant difference between the two groups (Table 1 and Table 2). The time interval between CT and echocardiography was 4.0 ± 5.5 days in the IE group and 3.0 ± 5.4 days in the control group (*p* = 0.46).

**Figure 6:**
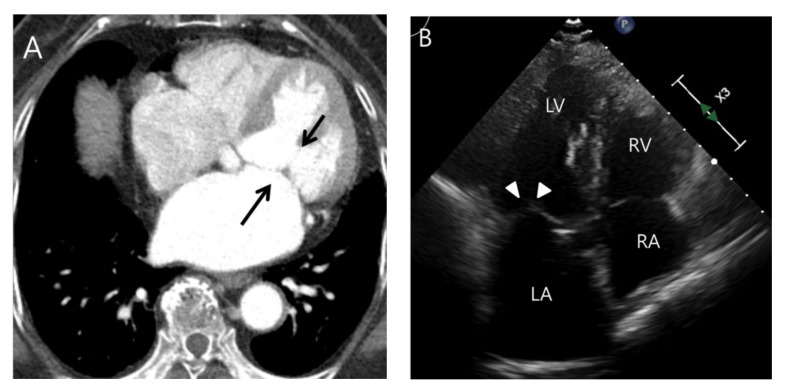
An example of a false positive diagnosis on routine chest CT due to motion artifact in an 84-year-old female patient. (**A**) Subtle nonelongated nodular thickening of the mitral valve is noted on an axial CT image (arrows). However, there was no splenic or renal infarction in this patient. (**B**) Subsequent echocardiography demonstrates negative findings (arrowheads).

**Figure 7:**
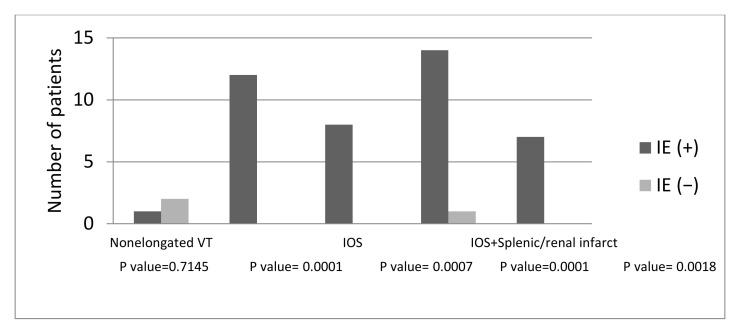
Differences in the incidence of the variable CT findings between study patients and control group. IE, VT, IOS indicate infective endocarditis, valve thickening, and the indirect-oscillation sign, respectively.

There was no significant difference in the incidence of nonelongated nodular valve thickening on CT between the study and control groups [3.7% (1/27) versus 5.7% (2/35), *p* = 0.7145] (Figure 6). In contrast, there was a significant difference of incidence of elongated nodular valve thickening on CT in those with IE as compared to the control group [44.5% (12/27) and 0% (0/35), *p* = 0.0001]. Notably, the sensitivity, specificity, PPN, and NPV for the indirect-oscillation sign on routine chest CT were 29.6% (8/27), 100% (35/35), 100% (8/8), and 64.8% (35/54), respectively (Table 3 and Table 4, Figure 7). Five patients had complications [abscess, *n* = 4; and abscess and pseudoaneurysm, *n* = 1] based on operative records (*n* = 14). Of these, one abscess and one pseudoaneurysm were identified on retrospective evaluation of the routine chest CT.

Splenic or renal infarction was noted in 51.9% (14/27) of the patients with IE and 3% (1/35) of the control group (*p* = 0.0001). Combining the indirect-oscillation sign and splenic or renal infarction findings resulted in 100% specificity (Table 3 and Table 4).

The interobserver agreement of positive findings for IE on routine chest CT was substantial (kappa value = 0.77). 

There was a significant difference in the echocardiographic diameter of nodular valve thickening (14.25 ± 5.7 mm vs. 8.9 ± 3.1 mm, *p* = 0.01) between patients with positive and those with negative CT findings. 

## 4. Discussion

Routine chest CT is not essential for the diagnostic workup for IE. However, non-ECG-gated routine chest CT may be the initial imaging modality in the evaluation of patients with IE presenting to the ED with nonspecific clinical symptoms. 

On echocardiography, the presence of a vegetation with oscillating motion is one of the major criteria for IE based on the modified Duke criteria [5]. Unfortunately, routine chest CT cannot directly assess the oscillating motion of a potential vegetation due to the lack of cine imaging data. However, based on recent clinical experience, the authors hypothesized that a combination of two particular findings on routine chest CT (the indirect-oscillation sign) might suggest the oscillating motion of the vegetation. These are (1) elongated nodular thickening; (2) abnormal orientation from the normal valve cups with a blind end. The main findings in this study were consistent with this hypothesis. 

Routine chest CT may be the initial imaging modality after chest radiograph used to evaluate IE if a patient presents to the ED with nonspecific fever or acute dyspnea. In such patients who present with vague clinical symptoms, suggesting IE on routine chest CT may be beneficial, leading to a more rapid diagnosis and proper management. Based on our findings, prospective studies validating the indirect-oscillation sign and conducting assessments using more-advanced CT technologies to improve its sensitivity may be potential areas for investigation. On routine chest CT in patients with documented IE, nonelongated nodular thickening involving the mitral or aortic valve was quite rare (3.7%, *n* = 1/27) as compared to elongated nodular thickening (44.4%, *n* = 12/27). This may reflect the fact that larger vegetations show an elongated shape rather than a nodular morphology and thus are more easily identified on routine chest CT. In support of this, the echocardiographic mean diameter of the vegetation (8.9 ± 3.1 mm) in false negative diagnoses was smaller than that of true positive diagnoses (14.3 ± 5.7 mm) on routine chest CT in this study [9]. Notably, one study showed that larger vegetations (greater than 10 mm) are associated with a higher risk for systemic arterial embolization. Thus, IE identified on routine chest CT may have meaningful clinical implications [18].

There were two cases of a false positive diagnosis of IE in the control group, both attributed to motion artifacts on routine chest CT. Thus, the presence of nonelongated nodular valve thickening may be a nonspecific finding, with the differential diagnosis including vegetation, benign tumor, or artifact. In contrast, the presence of elongated nodular thickening demonstrating an abnormal orientation from the normal aortic or mitral valve leaflets and having a blind end (i.e., an indirect sign of an oscillating vegetation) showed a high specificity (100%) and positive predictive value (100%), although a low sensitivity (29.6%). In this study, all cases with moderate-to-severe motion blurring on the routine chest CT were deemed negative for IE. This study design may have resulted in a lower sensitivity of the routine chest CT for the identification of IE. However, this is an appropriate approach in clinical practice as a high specificity and a positive predictive value for identifying IE on routine chest CT are valuable for patients in the ED with a low-to-intermediate pretest probability of IE. ECG-gated cardiac computed tomography (CT) has demonstrated its utility in preoperative planning for IE patients, allowing the visualization of coronary artery anatomy, the major cardiovascular structures, and the diagnosis of perivalvular complications such as pseudoaneurysm and abscess [19,20,21,22,23]. In this study, routine chest CT identified only 40% of patients with IE (*n* = 2/5) with a complication (abscess or pseudoaneurysm) confirmed at operation. False negative results for complications may have been driven by the small size of the complications and/or motion blurring. Based on this study, the ability to identify the complications of IE cannot be definitively assessed due to the small number of IE complications, and further evaluation is required to address this issue. 

CT has a wide *z*-axis coverage compared to echocardiography. This is an important relative strength in favor of CT, leading to the identification of extracardiac findings of IE such as splenic or renal infarction. For splenic or renal infarctions identified on routine chest CT, we found a low sensitivity (51.9%), high specificity (97.1%), high positive predictive value (93.3%), and modest negative predictive value (72.3%). The combination of the indirect-oscillation sign and splenic or renal infarction on routine chest CT resulted in high specificity and positive predictive values (100%). Thus, the presence of splenic or renal infarction in patients at the ED presenting with nonspecific fever or acute dyspnea is an important clue leading the to suspicion of IE on routine chest CT. Overall, radiologists should attempt to identify elongated thickening of the aortic or mitral valve, particularly if splenic or renal infarction is noted in patients at risk in the ED.

This study has several limitations. First, it is a retrospective study with a small number of patients. Second, there may be selection bias, as this study included only patients who underwent routine chest CT and echocardiography within one month of each other. Third, the diagnosis of IE was made by operative findings in only a small majority of patients (55.6%, *n* = 15/27). However, the diagnostic criteria for IE were met in all patients according to standard guidelines [8].

## 5. Conclusions

Although uncommon, we found that the combination of elongated nodular thickening and an abnormal orientation from the normal mitral valve with a blind end (the indirect-oscillation sign) showed high specificity and PPV. Identification of the indirect-oscillation sign involving the aortic or mitral valve on routine chest CT may assist in diagnosing patients with IE presenting to the ED with a nonspecific clinical presentation, leading to more rapid diagnosis and proper management. In addition, splenic or renal infarctions also showed high specificity and PPV. Thus, the presence of splenic or renal infarction in patients presenting to the ED with nonspecific fever or acute dyspnea is an important clue leading to the suspicion of IE on routine chest CT. Overall, radiologists should attempt to identify elongated thickening of the aortic or mitral valve particularly if splenic or renal infarction is noted in patients at risk in the ED.

## Figures and Tables

**Table 1:** Clinical and echocardiographic findings in the patient and control groups.

	IE (*n* = 27)	Control (*n* = 35)	*p*
Sex (M/F)	16/11	13/22	0.09
Mean age	56.3 ± 15.7	63 ± 13.9	0.08
Fever	16	15	0.20
Dyspnea	17	18	0.36
Sepsis	5	3	0.28
Hypertension	10	15	0.64
DM	6	11	0.42
CT–echocardiography interval, days	4.0 ± 5.5	3.0 ± 5.4	0.46
CT–surgery interval, days	9.8 ± 9.6 (*n* = 12)	0	
IE involvement site			
Aortic valve	8	0	
Mitral valve	14	0	
Both (aortic and mitral)	5	0	
Bicuspid aortic valve	5	0	0.01
Splenic or renal infarction	12	1	0.0001

**Table 2:** Results of blood cultures.

	Infective Endocarditis (*n* = 27)	Control (*n* = 35)
Staphylococci	6	0
Streptococci	5	0
Enterococci	3	0
Others	4	1
Negative	9	34

**Table 3:** Diagnostic accuracy of routine chest CT for the diagnosis of infective endocarditis per patient.

	Sensitivity	Specificity	PPV	NPV
Per-patient				
Nonelongated VT	1/27 (3.7)	33/35 (94.3)	1/3 (33.3)	33/59 (57.9)
Elongated VT	12/27 (44.5)	35/35 (100)	12/12 (100)	35/50 (70)
Indirect-oscillation sign	8/27 (29.6)	35/35 (100)	8/8 (100)	35/54 (64.8)
Splenic or renal infarct	14/27 (51.9)	34/35 (97.1)	14/15 (93.3)	34/47 (72.3)
Indirect-oscillation sign + splenic or renal infarct	7/27 (25.9)	35/35 (100)	7/7 (100)	35/55 (63.6)

Percentage in parentheses. VT indicates valve thickening. PPV and NPV indicate positive and negative predictive values, respectively.

**Table 4:** Diagnostic accuracy of routine chest CT for the diagnosis of infective endocarditis per valve.

	Sensitivity	Specificity	PPV	NPV
Per valve (aortic valve)				
Nonelongated VT	0/13 (0)	49/49 (100)	0/0 (0)	49/62 (79)
Elongated VT	4/13 (30.8)	49/49 (100)	4/4 (100)	49/58 (84.5)
Indirect-oscillation sign	1/13 (7.7)	49/49 (100)	1/1 (100)	49/61 (80.3)
Splenic or renal infarct	9/13 (69.2)	48/49 (98)	9/10 (90)	48/52 (92.3)
Indirect-oscillation sign + splenic or renal infarct	1/13 (7.7)	49/49 (100)	1/1 (100)	49/61 (80.3)
Per valve (mitral valve)				
Nonelongated VT	1/19 (5.3)	41/43 (95.3)	1/3 (33.3)	41/59 (69.5)
Elongated VT	8/19 (42.1)	43/43 (100)	8/8 (100)	43/54 (79.6)
Indirect-oscillation sign	7/19 (36.8)	43/43 (100)	7/7 (100)	43/55 (78.2)
Splenic or renal infarct	10/19 (52.6)	42/43 (97.7)	10/11 (90.9)	42/51 (82.4)
Indirect-oscillation sign + splenic or renal infarct	6/19 (31.6)	43/43 (100)	6/6(100)	43/56 (76.8)

Percentage in parentheses. VT indicates valve thickening. PPV and NPV indicate positive and negative predictive value, respectively.

## Data Availability

The data provided in this study can be obtained by contacting the corresponding author. Due to ethical considerations, the data are not available for public access.

## Abbreviations

IE: infective endocarditis; TTE, transthoracic echocardiography; TEE, transesophageal echocardiography; CT, computed tomography; ED, emergency department; MDCT, multidetector CT; NPV, negative predictive value; PPV, positive predictive value; TP, true positive; FN, false negative; TN, true negative; FP, false positive.
